# Sterilization of New Endodontic Hand Files Stored in Dental Office Inventory: An In Vitro Study

**DOI:** 10.7759/cureus.36116

**Published:** 2023-03-14

**Authors:** Surender LR, Sravani Nirmala, Narender Reddy, Rakesh Reddy Chukka, Sainath D Reddy, Naresh Kumar K

**Affiliations:** 1 Conservative Dentistry and Endodontics, SVS Institute of Dental Sciences, Mahabubnagar, IND

**Keywords:** nutrient broth, turbidity, bacteria, macckonkey agar plates, blood agar plates, sterilization, blisters, boxes, root canal files, microorganisms

## Abstract

Background and objective

Endodontic files, as supplied by the manufacturers to the endodontists, are not pre-sterile routinely. For both new and used equipment, rotary as well as manual, autoclaving is the standard sterilization protocol used in clinical and academic practice. In dentistry, instrument sterilization aims to safeguard patients from cross-contamination through instruments. Hence, every device should be thoroughly cleaned and sterilized. In this study, we aimed to evaluate the presence of various microorganisms in sealed and unsealed stored packs in dental offices and the probable impact of pre-sterilization procedures on the survival of these microorganisms.

Materials and methods

Two groups of root canal files varying in their packing method, boxes (Mani stainless steel K-files, ISO 25, length 25 mm) and blister packs (UGD, ISO 25, length 25 mm), pre-sterile, opened/unopened were chosen and stored for about two weeks in the dental office and were classified into three groups based on their storage and further classified into subgroups depending on their packing modes as follows: Group-1 (unopened and stored in shelf for two weeks), Subgroup-1A (boxes), Subgroup-1B (blister packs); Group-2 (unopened and stored on the countertop for two weeks), Subgroup-2A (boxes), Subgroup-2B (blister packs); Group-3 (opened and stored on the countertop for two weeks). After two weeks of storage, a set of three new files from each pack, both boxes and blisters, were placed in the nutrient broth to assess the turbidity and later cultured to assess the presence/absence and type of any bacterial growth. All the instruments in the three groups and subgroups were placed separately in the nutrient broth and carried to the microbiology lab for bacterial cultures. The entire procedure was carried out under the laminar flow. All these files in the nutrient broth were incubated for about 72 hours and the turbidity was assessed, and then the turbid bacteria were cultured on blood agar and MacConkey agar plates for the presence/absence and the type of bacteria in each group and subgroups.

Results

All specimens, both opened/unopened boxes and blister packs, after about two weeks of storage, were cultured and observed for contamination. All the tested files groups showed bacterial culture growth both on blood agar and MacConkey agar plates. Group-1 (Subgroups 1A, 1B) boxes and blister packs unopened and stored on the shelf for two weeks demonstrated aerobic spore bacilli; Group-2 (Subgroups 2A, 2B) boxes and blister packs unopened and stored on the countertop for two weeks demonstrated Gram-positive bacilli; Group-3 opened boxes and blisters stored on the countertop for two weeks demonstrated Micrococci and Gram-negative bacilli.

Conclusion

In the present study, all the packs, blisters and boxes, demonstrated the presence of bacterial growth irrespective of their storage in the dental office. Hence, in order to prevent any new infections from the operating field, sterilization of not only the old files but also the pre-sterilization of new files should be made mandatory.

## Introduction

Microorganisms cause a variety of contagious diseases in the human body. Contamination leads to the transmission of infectious agents, and hence infection control is of paramount concern in the medical and dental healthcare environments. In dentistry, it is essentially linked to the processing of reusable instruments to prevent cross-infection. In endodontics, various instruments are used to clean and shape the root canal, as well as to eliminate microbial load in the root canal space. These instruments may directly come into contact with tissues, blood and tissue fluids, saliva, and gingival crevicular fluid. Sterilization can impede the spread of infectious diseases. In the present scenario, the universal norm is as follows: if you can sterilize an instrument, sterilize it, or dispose of it. There are several methods to sterilize these instruments, which include dry heat sterilization, autoclaving, and the use of ethylene oxide gas, glass-bead sterilizer, hot-salt sterilizer, etc. [1,2].

Precleaning and sterilization of some devices can be difficult because of their small size and complex architecture. Endodontic files are slender, tapered instruments, about 21-, 25-, and 31-mm long, with intricate topography and spiral cutting edges used for cleaning and shaping root canals during endodontic treatment. Because of their size and shape, it is difficult to remove all biological material during re-sterilization procedures [2].

Undoubtedly, the major factors associated with endodontic failure are the persistence of microorganisms in the root canal space and/or the periapical tissues. The dentist is often deceived by the notion that procedural mishaps, such as broken instruments, perforations, overfilling, underfilling, ledges, and so on, are the definitive cause of endodontic failure. Root canal treatment usually has a poor prognosis when treatment falls short of adequate standards [3]. While the entire world is looking to abolish existing infectious diseases, it is of utmost importance to prevent or minimize any new infection from the operating field or the armamentarium. Endodontic files, as supplied by the manufacturers, are not pre-sterile routinely [1,2].

While we may not completely succeed in preventing infections, we can surely minimize the occurrence of new infections, which is the least observed item in the failure list of an unsuccessful endodontic procedure. Not only the sterilization of old instruments but also the pre-sterilization of packed new instruments should be prioritized to minimize infections [2,4].

There are scarce data in the literature on this topic. Hence, in this study, we aimed to analyze the phenomenon of bacterial colonization on unused packed instruments. The study was undertaken to evaluate the presence of various microorganisms in sealed and unsealed stored packs depending on different environmental conditions in dental clinics and the probable impact of pre-sterilization on the survival of these microorganisms.

## Materials and methods

The study was conducted in the Department of Conservative Dentistry and Endodontics, SVS Institute of Dental Sciences, Mahabubnagar. The bacterial cultures were done in the Department of Microbiology, SVS Institute of Medical Sciences, Mahabubnagar.

Three groups of root canal files, comprising a total of 10 files (Mani stainless steel K-files, ISO 25, length 25 mm), two sets of blister packs (UGD, ISO 25, length 25 mm) with a total of 10 files (unopened/opened) were chosen and stored for about two weeks and were classified into three groups with two subgroups based on their package mode as follows:

Group-1 (unopened and stored on the shelf for two weeks); boxes and blister packs; Subgroup-1A included a set of three new files from boxes that were placed in the nutrient broth; Subgroup-1B included a set of three new files from blister packs that were placed in the nutrient broth.

Group-2 (unopened and stored on the countertop for two weeks); boxes and blister pack; Subgroup-2A included a set of three new files from boxes that were placed in the nutrient broth; Subgroup-2B included a set of three new files from blister packs that were placed in the nutrient broth.

Group-3 (opened and stored on the countertop for two weeks) included a set of six files from boxes and blister packs that were placed in the nutrient broth.

**Figure 1:**
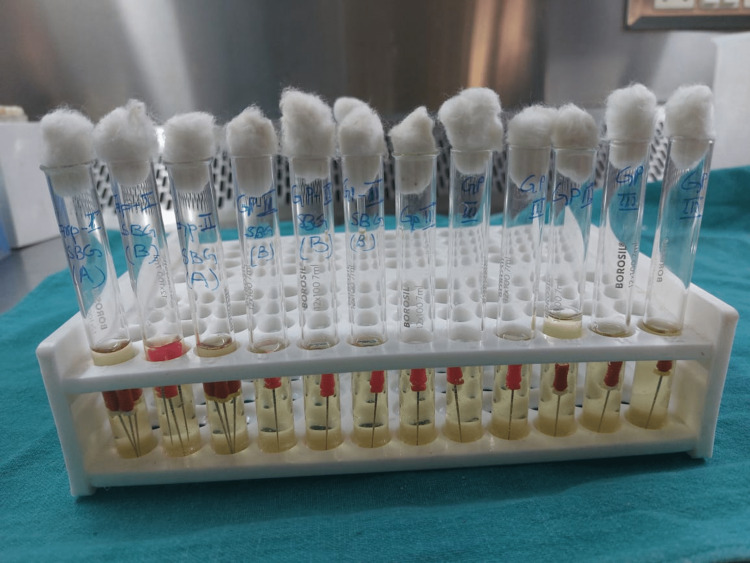
Group-1, Group-2 (Subgroups 1A, 1B, 2A, 2B), and Group-3 files placed in the nutrient broth after two weeks of storage

After about two weeks of storage, a set of three new files from each pack, both boxes and blisters, were placed in the nutrient broth (Figure 1) to assess the turbidity and were later cultured to assess the presence/absence and type of any bacterial growth. All the instruments in the three groups and subgroups were placed separately in the nutrient broth and carried to the microbiology lab for bacterial cultures.

**Figure 2:**
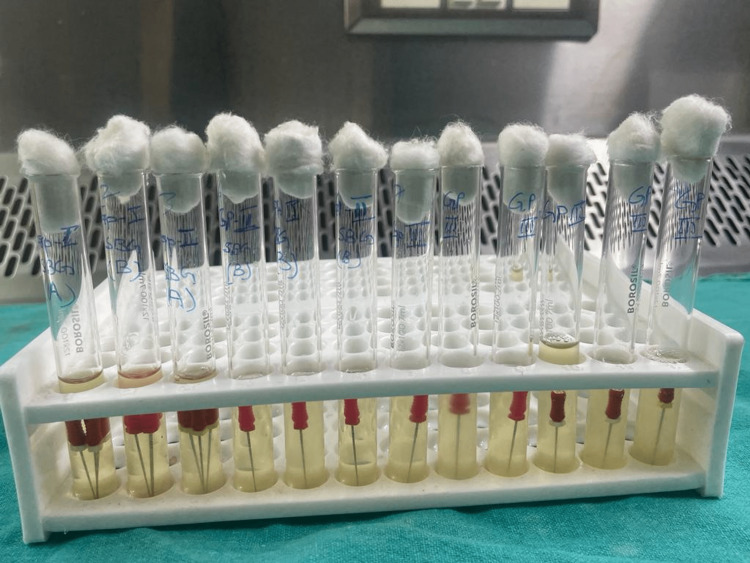
Turbidity in the nutrient broth evaluated after 72 hours

All these files in the nutrient broth were incubated for about 72 hours and the turbidity was assessed (Figure 2), and then the turbid bacteria were cultured on blood agar and MacConkey agar plates for the presence/absence and the type of bacteria in each group and subgroups. The entire procedure was carried out under the laminar flow.

## Results

All the tested file groups showed bacterial culture growth both on blood agar and MacConkey agar plates.

**Figure 3:**
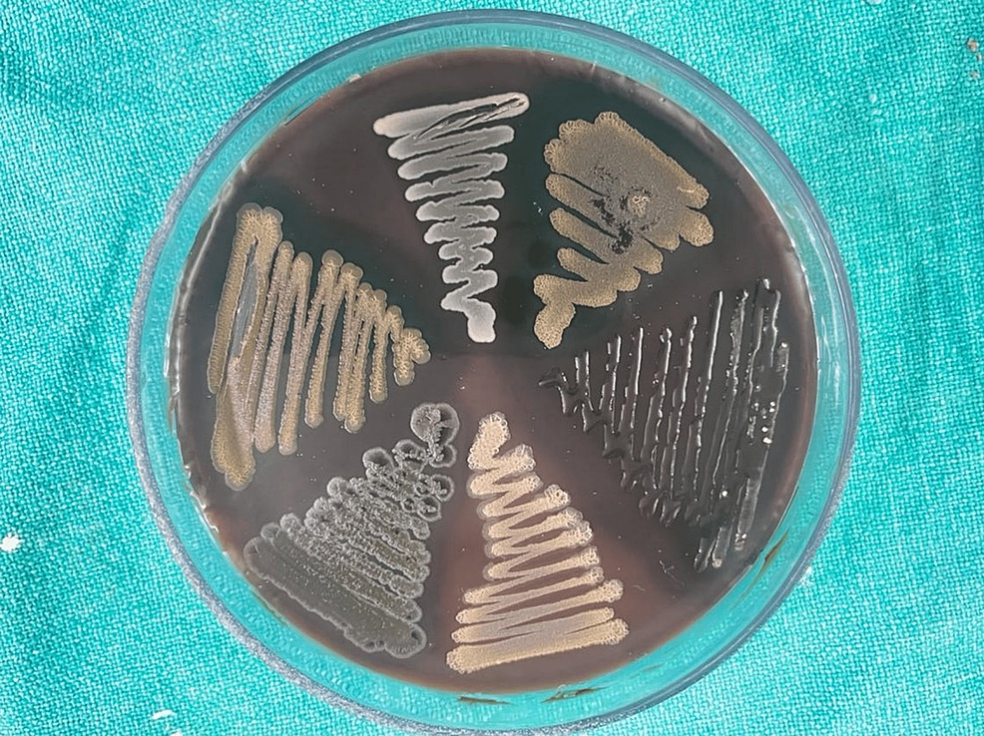
Bacterial culture growth on blood agar plates

Group-1: Subgroups (1A,1B) demonstrated the following bacterial cultures on blood agar plates, which are aerobic spore bacilli (Figure 3).

**Figure 4:**
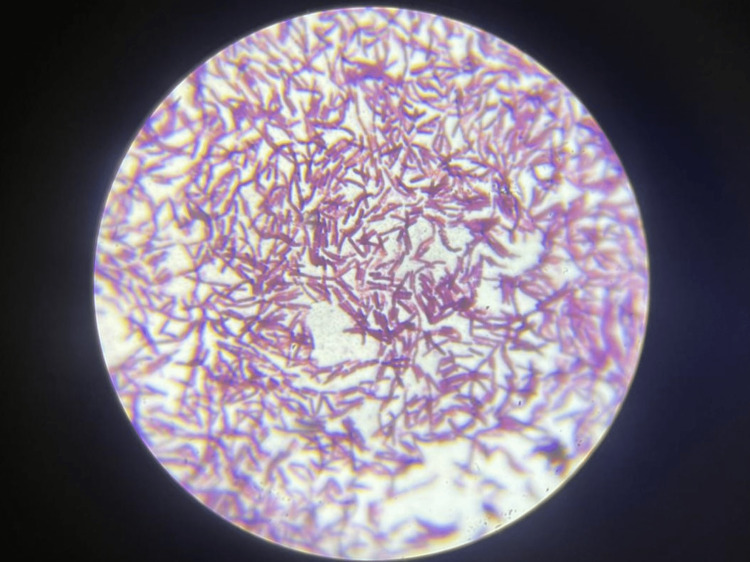
Microscopic image of aerobic spore bacilli

The microscopic image depicting aerobic spore bacilli is shown in Figure 4.

**Figure 5:**
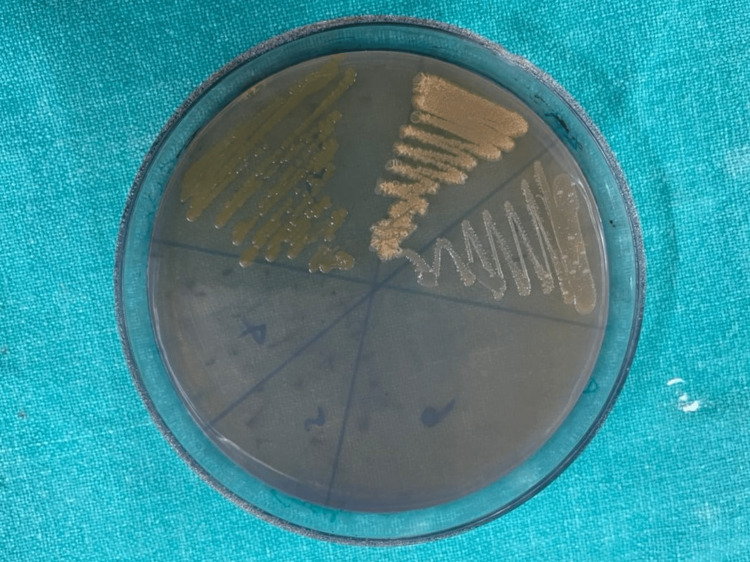
Bacterial culture growth on MacConkey agar plates

Group-2: Subgroups demonstrated the following bacterial cultures on blood agar plates, which are aerobic spore bacilli (Figure 3), and MacConkey agar plates showed Gram-positive cocci (Figure 5).

**Figure 6:**
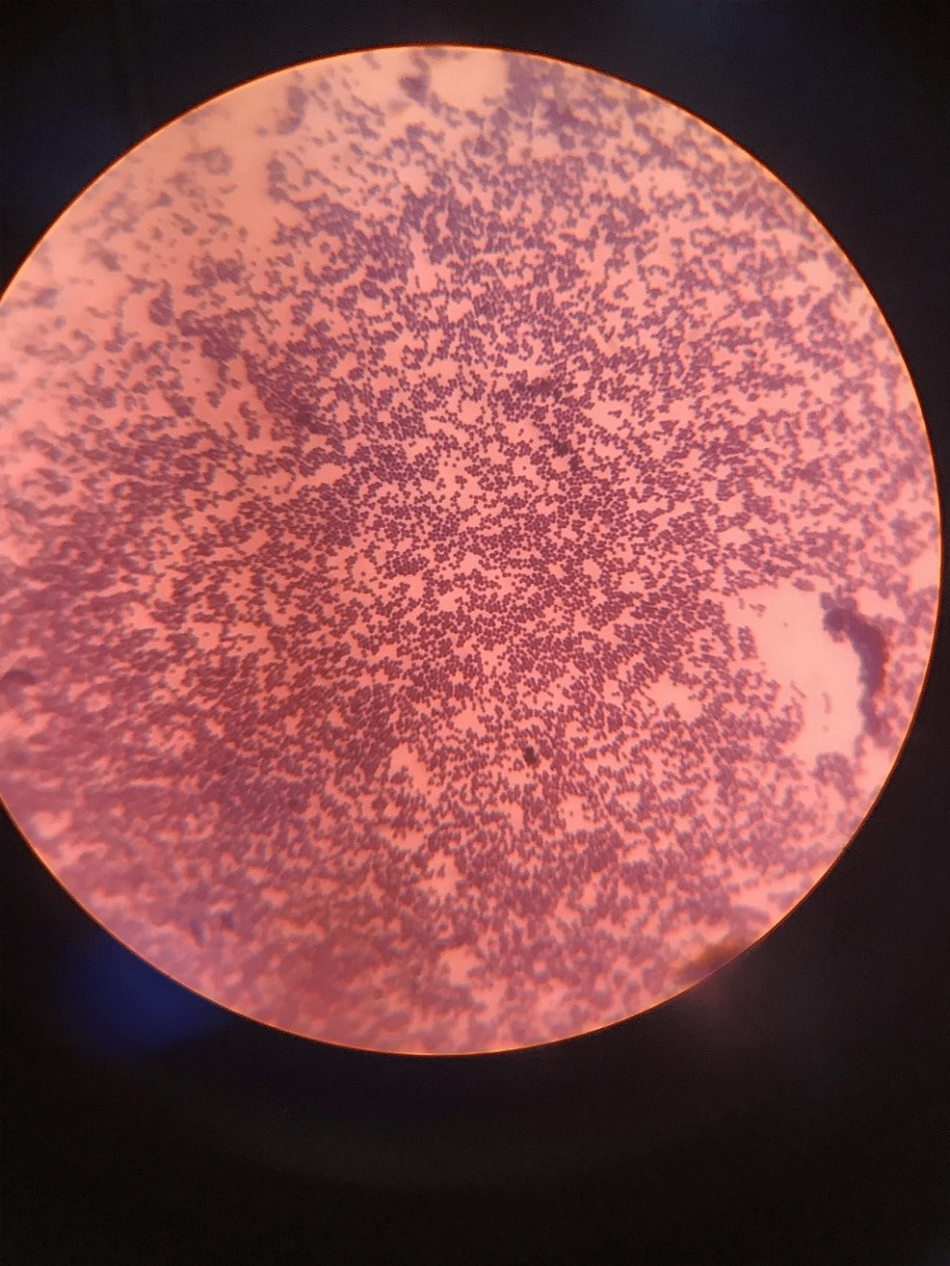
Microscopic image of Gram-positive cocci

The microscopic image depicting Gram-positive cocci is shown in Figure 6.

**Figure 7:**
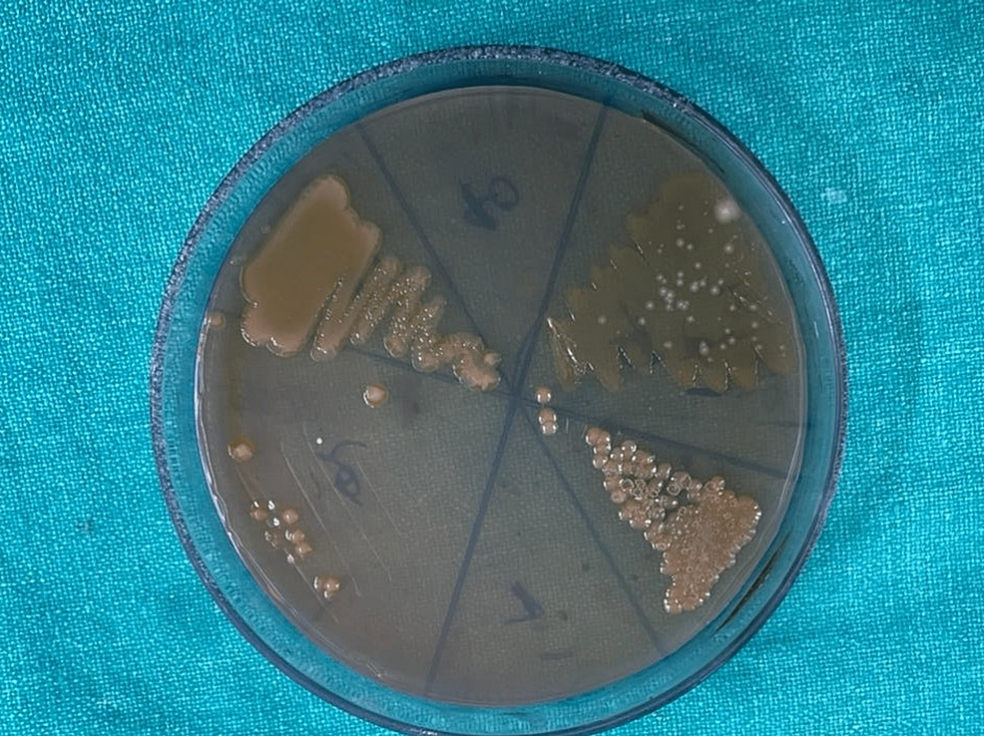
Bacterial culture growth on MacConkey agar plates

Group-3 demonstrated the following bacterial cultures on blood agar plates, which are aerobic spore bacilli (Figure 3), and Gram-positive cocci and Gram-negative bacilli on MaCconky agar plates (Figure 7).

**Figure 8:**
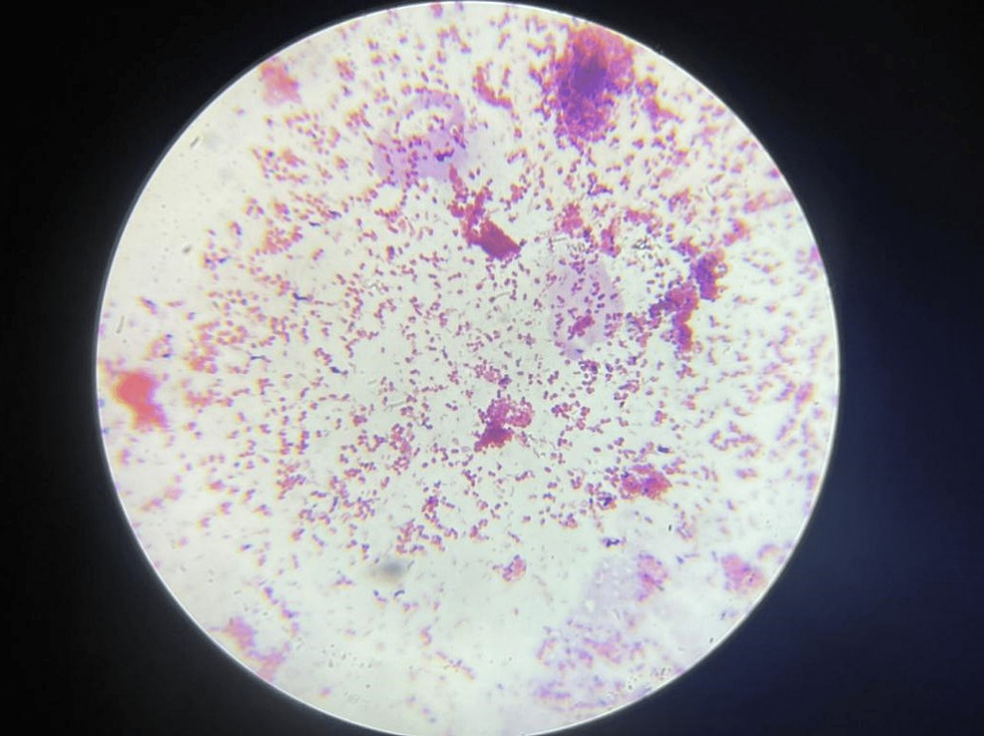
Microscopic image of Gram-negative bacilli

The microscopic image depicting Gram-negative bacilli is shown in Figure 8.

## Discussion

Infection control is a major concern in medicine and dentistry because of the transmissibility of infectious diseases. Additionally, asepsis is paramount in endodontics because microorganisms are the primary cause of endodontic diseases [5]. The classic triad for the success of endodontic treatment comprises canal instrumentation, disinfection, and obturation, of which canal instrumentation is accomplished by endodontic files. Thus, the sterility of instruments is imperative for success in endodontic treatment [6].

In the present study, Group-1 (Subgroups 1A, 1B) and Group-2 (Subgroups 2A, 2B) demonstrated the presence of aerobic spore bacilli (Figures 3, 4) and Gram-positive cocci (Figures 5, 6). The presence of these bacteria may be due to their ability to survive and form biofilms on the stainless steel equipment, which indicates the contamination of the stored samples [7]; similar results were seen in a study that concluded that the new or disposable instruments must be decontaminated before use because they may present a certain degree of bacterial contamination such as cocci [2,4].

Similar results were seen in another study that demonstrated the presence of microorganisms (P. lentimorbus). In the same study, they demonstrated the presence of metal residues such as chromium-nickel residues (present on the surface from the production processes) [6]. These first-use instruments must necessarily undergo a cleansing and decontamination phase to remove metal residues by an ultrasonic bath or a disinfected tray and with a few minutes of immersion in disinfectant solutions such as 2% chlorhexidine or 2% peracetic acid [6].

Group-3 demonstrated the presence of Gram-positive cocci (Figures 5, 6) and Gram-negative bacilli (Figures 7, 8). Every patient should be treated as potentially vulnerable to infections [8]. Strict aseptic principles need to be incorporated into clinical practice in order to reduce microbial cross-contamination. For minimizing cross-contamination, different materials and procedures are recommended, such as the use of personal protective barriers, decontamination of surfaces, immunization of dental staff, sterilization of instruments, and pre-procedural mouthwashes [9].

The files that were unopened and stored on the shelf demonstrated the presence of non-pathogenic bacteria and the files that were sealed and kept on the countertop in the dental office demonstrated the presence of Gram-positive cocci. Gram-positive cocci can cause inflammatory diseases, including skin infections, pneumonia, endocarditis, septic arthritis, osteomyelitis, and abscesses [10]. The files that were opened and kept on the countertop of the dental office demonstrated Gram-positive cocci and Gram-negative bacilli. Gram-negative bacteria cause infections including pneumonia, bloodstream infections, wound or surgical site infections, and meningitis in healthcare settings [11].

Based on our findings, it is imperative that disposable or first-use instruments should be pre-sterilized prior to use, both to remove contamination by microorganisms and to get rid of any residue from the instruments. A study has concluded that the adoption of a combination of mechanical, chemical, and ultrasonic cleaning methods as a standard protocol can provide complete cleaning of biological debris from endodontic instruments. All the endodontic files should be cleaned ultrasonically to remove the surface residue followed by autoclave procedures, which are considered gold standard sterilization procedures, and packed and stored to prevent cross-contamination by pathogenic bacterial species [5-6,12-14].

## Conclusions

Endodontic files, as supplied by the manufacturers, are not pre-sterile routinely. The ultimate goal of instrument sterilization in dentistry is to safeguard patients from cross-infection by instruments. In the present study, even the new files were contaminated with bacteria, showing the importance of pre-sterilization procedures. Thus, utmost care should be taken to clean and disinfect every instrument prior to its use in patients.
